# Alteration of the Chicken Upper Respiratory Microbiota, Following H9N2 Avian Influenza Virus Infection

**DOI:** 10.3390/pathogens12091168

**Published:** 2023-09-16

**Authors:** Tara Davis, Dagmara Bialy, Joy Leng, Roberto La Ragione, Holly Shelton, Klaudia Chrzastek

**Affiliations:** 1 The Pirbright Institute, Pirbright, Woking GU24 0NF, UK; tfd26@mrc-tox.cam.ac.uk (T.D.); dagmara.bialy@pirbright.ac.uk (D.B.); holly.dove@hse.gov.uk (H.S.); 2 School of Veterinary Medicine, University of Surrey, Guildford GU2 7XH, UK; joy.leng2@liverpool.ac.uk (J.L.); r.laragione@surrey.ac.uk (R.L.R.); 3 Institute of Integrative Biology, University of Liverpool, Liverpool L69 3BX, UK; 4 School of Biosciences, University of Surrey, Guildford GU2 7XH, UK; 5 Animal and Plant Health Agency, Pathology and Animal Sciences, APHA, Addlestone KT15 3NB, UK

**Keywords:** microbiota, respiratory tract, influenza, H9N2, sequencing, chicken

## Abstract

Several studies have highlighted the importance of the gut microbiota in developing immunity against viral infections in chickens. We have previously shown that H9N2 avian influenza A virus (AIV) infection retards the diversity of the natural colon-associated microbiota, which may further influence chicken health following recovery from infection. The effects of influenza infection on the upper respiratory tract (URT) microbiota are largely unknown. Here, we showed that H9N2 AIV infection lowers alpha diversity indices in the acute phase of infection in the URT, largely due to the family Lactobacillaceae being highly enriched during this time in the respiratory microbiota. Interestingly, microbiota diversity did not return to levels similar to control chickens in the recovery phase after viral shedding had ceased. Beta diversity followed a similar trend following the challenge. *Lactobacillus* associate statistically with the disturbed microbiota of infected chickens at the acute and recovery phases of infection. Additionally, we studied age-related changes in the respiratory microbiota during maturation in chickens. From 7 to 28 days of age, species richness and evenness were observed to advance over time as the microbial composition evolved. Maintaining microbiota homeostasis might be considered as a potential therapeutic target to prevent or aid recovery from H9N2 AIV infection.

## 1. Introduction

Avian influenza A viruses (AIVs) belong to the *Orthomyxoviridae* virus family and have a single-stranded, negative-sense RNA genome [1]. AIVs can be categorised into high- and low-pathogenic viruses (HPAIVs and LPAIVs, respectively), depending on their disease severity [2]. HPAIVs have up to 100% mortality rates and infect chickens systematically, whereas LPAIVs are associated with milder symptoms and lower mortality rates and are limited to respiratory and intestinal tracts [3]. This difference in pathogenicity is determined by the haemagglutinin (HA) protein, based on which proteases recognise the proteolytic cleavage site sequence [1]. The H9N2 subtype was first identified in China in the early 1990s and is now considered to be endemic in poultry throughout Asia, Europe, North Africa and the Middle East [4,5,6,7]. Despite the H9N2 subtype generally having a mortality rate of less than 20%, outbreaks are associated with decreased egg production and clinical symptoms, as well as an increased likelihood of secondary infections with potentially higher mortality rates [8,9]. It is also considered zoonotic, as it can be transferred to humans and can also be transferred to other mammals, such as pigs [8,10]. Therefore, being able to treat and control the spread of H9N2 AIV in poultry populations is important for preventing economic losses and protecting public health.

There is growing evidence that the commensal bacteria present at the body sites of chickens play an important role in shaping the host’s defences against viral infection [11]. Variations in the gut microbiome composition have been shown to be caused by viral infection, as well as environmental factors like housing, diet and hygiene [12,13]. The relationship between the gut microbiota and AIV in the chicken model has been observed in numerous studies [14,15,16,17]. These studies have shown that H9N2 AIV infection is associated with the dysregulation of the gut microbiota, as well as changes in innate immune gene expression levels. Yitbarek et al. (2018) showed that the depletion of the gut microbiota by a cocktail of antibiotics leads to a significant downregulation of the interleukin-22 (IL-22) [16]. IL-22 is an important cytokine in the gastrointestinal tract, as it plays a role in maintaining homeostatic levels and host defences against microbes, particularly in barrier repair [18]. However, when the gut microbiota is restored using probiotics or faecal matter transplants (FMTs), the levels of IL-22 were restored to levels similar to chickens that had not undergone depletion of their gut microbiota [16], thus demonstrating the key part that the gut microbiota plays in viral pathogenesis. We have previously shown reduced colon microbiota alpha diversity in the acute period of AIV infection (days 2–3) in both Rhode Island Red and VALO chicken lines which did not reach the same level as in uninfected chickens by day 10 post infection, which may further influence chicken health following recovery from infection [19]. 

Compared to the gut microbiota, the respiratory microbiota has been less well studied. It has been shown that the composition of the respiratory microbiota in chickens is also altered by host-related and environmental factors, such as age, temperature and farm location, but little is known about the relationship between the respiratory microbiome and viral infection in chickens [20,21,22]. LPAIVs have been shown to have a tropism for the respiratory tract and replicate there extensively [11,23,24]. Therefore, it is important to understand how the microbiota in the respiratory tract of chickens is altered following influenza A virus infection, as this may have an impact on the innate immune responses elicited there and the resulting susceptibility to infection. Understanding these changes in the respiratory microbiota could provide information useful for the development of disease prevention and treatment strategies in the future.

In this study, Rhode Island Red (RIR) chickens were used to assess temporal changes in the chicken upper respiratory tract (URT) microbiota following H9N2 AIV infection. Additionally, the study aimed to assess how the healthy chicken respiratory microbiota changes during maturation up to 28 days of age.

## 2. Materials and Methods

### 2.1. Experiment Design

In this study, two in vivo animal experiments were performed. In Experiment 1, we used 35 specific pathogen-free (SPF) Rhode Island Red (RIR) chickens to assess the effect of H9N2 AIV infection on upper respiratory tract (URT) microbiota. These birds were reared together until two weeks of age when they were randomly separated into two groups: the control (*n* = 20) and H9N2-AI-infected groups (*n* = 15). At three weeks of age, the infected birds received 100 µL of 10^5^ plaque-forming units (pfu/mL) recombinant H9N2 A/chicken/Pakistan/UDL01/01 via the intranasal route (50 µL in each nostril). On the day of challenge, 5 birds were randomly selected from the control group and culled to establish starting microbiota profile. On days 2, 4 and 10 post-challenge (p-ch), trachea samples were taken from 5 birds from both the control and infected groups. In Experiment 2, 20 SPF RIR chickens were kept in a cage together, and 5 birds were randomly selected and culled at days 7, 14, 21 and 28 of age to assess URT microbiota changes over time. These data were then compared with URT microbiota results obtained from Experiment 1 to further understand the changes that occurred during infection. RIR chickens were provided as day-old chicks from the National Avian Resource Facility (NARF) located at the Roslin Institute, Edinburgh, UK. The feed was provided ad libitum according to manufacturer’s instructions for the chicken age. All chicks moved from starter feed to grower at 3 weeks old. Control groups were housed in raised-floor pens whilst AIV-challenged chickens were housed in self-contained BioFlex^®^ B50 Rigid Body Poultry isolators (Bell Isolation Systems, Livingston, UK) maintained at negative pressure. All birds were swabbed daily from day of challenge until 8 days post infection to determine viral shedding. Swabbing was carried out with sterile polyester tipped swabs (Fisher Scientific, Loughborough, UK) which were transferred into viral transport media [24], vortexed briefly, clarified by centrifugation and stored at −80 °C prior to virus detection. The URT samples, included anatomically: cranial larynx from proximal part, distally to syrinx, up to part where trachea divides into bronchi. Each trachea was then cut out from larynx with sterile scalpel. Proximal part of trachea samples was used for DNA/RNA extraction and snap-frozen, whereas distal trachea was used for histology examination and thus fixed in 4% buffered formaldehyde.

### 2.2. Virus Propagation

Recombinant A/chicken/Pakistan/UDL01/08 H9N2 virus was generated using reverse genetics as previously described [25]. Virus stocks were produced via passage in 10-day-old embryonated chicken eggs; the allantoic fluid was harvested after 48 h and titrated by plaque assay on MDCK cells (ATCC). Madin-Darby Canine Kidney (MDCK) cells (ATCC) were maintained in DMEM (Gibco-Invitrogen, Inc., Waltham, MA, USA) supplemented with 10% fetal bovine serum (Biosera, Inc., Cholet, France), 1% penicillin/streptomycin (Sigma-Aldrich, Inc., St. Louis, MO, USA) and 1% non-essential aa (Sigma-Aldrich, Inc., St. Louis, MO, USA).

### 2.3. DNA Extraction and 16S rRNA Gene Amplification

Proximal part of trachea samples was cut and disrupted in the TissueLyser II (QIAGEN, Hilden, Germany) to homogenise the tissue. Each sample was then transferred to the PowerBead tubes provided in the DNeasy PowerSoil Kit (QIAGEN). These tubes were placed in the TissueLyser LT (QIAGEN) for next 10 min at 40 1/s. From this point onwards, the manufacturer’s instruction for the extraction of microbial genomic DNA using the DNeasy PowerSoil Kit was followed. Controls for DNA extraction reagents (negative control) and *E. coli* DH5α (Thermo Fisher, Waltham, MA, USA) (positive control) were included. DNA from both controls followed the same DNA extraction protocol as described above. Controls were included to assess the level of possible contaminations and robustness of the method. The V2-V3 region of the 16S rRNA gene was amplified via a two-step nested PCR, using the protocol described by Glendinning et al. [19]. This involved a V1-V4 region amplification using the primers 28F [5′-GAGTTTGATCNTGGCTCAG-3′] and 805R [5′-GACTACCAGGGTATCTAATC-3′]. This was followed by a short-cycle V2-V3 amplification using the primers 104F [5′-GGCGVACGGGTGAGTAA-3′] and 519R [5′-GTNTTACNGCGGCKGCTG-3′] with unique barcodes and Illumina adaptor sequences to prepare the gene segments for sequencing.

### 2.4. RNA Extraction and qRT-PCR

The supernatant from homogenised trachea tissue obtained at days 2 (*n* = 5) and 4 (*n* = 3) post-challenge was used for RNA extraction, using the QIAmp viral RNA minikit (Qiagen, Hilden, Germany) according to manufacturer instructions. For H9N2 influenza virus detection in trachea, quantitative analyses of matrix (M) were performed with primers IAV_F (5′-AGA TGA GTC TTC TAA CCG AGG TCG-3′) and IAV_R (5′-TGC AAA AAC ATC TTC AAG TCT CTG-3′) with IAV_probe (FAM-5′ TCA GGC CCC CTC AAA GCC GA-TAMRA-3′). qRT-PCR analysis was completed using the Superscript III Platinum one-step qRT-PCR kit (Life Technologies, Carlsbad, CA, USA). T7 RNA polymerase-derived transcripts from UDL-01 segment 7 were used for preparation of the standard curve. Cycling conditions were (1) 5 min at 50 °C, (2) a 2 min step at 95 °C and (3) 40 cycles of 3 s at 95 °C, 30 s of annealing and extension at 60 °C. Cycle threshold (CT) values were obtained using 7500 software v2.3.

### 2.5. Histology

Trachea samples from controls were taken from a range of birds on days 2–10 (N = 6), whereas samples collected from H9N2-infected birds were taken at days 2, 4 and 10 post-challenge (5 samples at each time point tested). These samples were fixed in 4% buffered formaldehyde. In total, 21 samples were commercially prepared by ProPath^®^ UK Limited (Hareford, UK). This included 15 samples from H9N2-infected group, five at each time point tested, accompanied by control samples, 2 at each time point tested. The tissues from 21 chickens were trimmed into cassettes as per Propath’s block list and Propath’s SOPs. The tissues were processed on a Tissue Tec VIP processor, dehydrated through a series of graded alcohols, cleared in toluene and infiltrated with poly wax. The processed tissues were then embedded in poly wax and sections cut at a nominal 4 µm, tissue sections were then placed on microscope glass slides. Slides were baked overnight in a 37 °C oven prior to staining. Slides from each of the 21 chicken trachea samples were dewaxed in xylene, hydrated through a series of graded alcohols and rinsed in running tap water. The slides were then stained in Mayer’s haematoxylin and washed in running tap water. The slides were then counter-stained with aqueous eosin and washed in two changes of running tap water. Finally, the slides were dehydrated through a series of graded alcohols, cleared in xylene and mounted with Pertex^®^. All stained slides from each of the 21 chickens were macroscopically and microscopically quality-checked by ProPath^®^ UK Limited (Hareford, UK) for the following acceptance criteria: satisfactory staining and cover slipping; presence of correct tissue/tissue area on slide, absence of section artefact, e.g., scores, creases or chatter; the presence of lesions and abnormalities noted at necropsy; and consistency of embedding.

Delafield’s haematoxylin- and eosin-stained slides were then analysed using a light microscope. The percentage of the inner epithelial layer that had either been degraded or had the cilia on its surface stripped away was estimated independently by two people, and the mean percentage damage of the two estimates was used in the statistical analysis. Two-tailed *t*-tests were used to assess significant differences between the control and infected groups at each time point * *p* < 0.05.

### 2.6. 16S rRNA Sequencing and Data Analysis

Libraries were analysed on a High Sensitivity DNA Chip on the Bioanalyzer (Agilent Technologies, Santa Clara, CA, USA) and Qubit dsDNA HS assay (Invitrogen, Waltham, MA, USA) and then loaded on the flow cell of the 500 cycle MiSeq Reagent Kit v2 (Illumina, Santa Clara, CA, USA) and pair-end sequencing (2 × 250 bp). Quantitative Insights into Microbial Ecology (QIIME) platform version qiime2-2019.10 was used to analyse sequencing dataset. Low-quality sequencing reads were quality-trimmed and denoised using DADA2 (trim position 15 for both forward and reverse sequencing reads generated). Potential chimeric sequences were removed using UCHIME, and the remaining reads were assigned to 16S rRNA operational taxonomic units (OTUs) based on 97% nucleotide similarity with the UCLUST algorithm and then classified taxonomically using the SILVA reference database (silva132-99-nb-classifer). Taxonomy was then collapsed to the genus level. The microbial community structure was estimated by microbial biodiversity (i.e., species richness and between-sample diversity). Shannon index, phylogenetic diversity and the observed number of species were used to evaluate alpha diversity, and the unweighted UniFrac distances were used to evaluate beta diversity. All these indices (alpha and beta diversity) were calculated using the QIIME pipeline.

### 2.7. Statistical Analysis of Sequencing Data

Kruskal–Wallis pairwise statistical testing was used to assess the differences in alpha diversity in terms of the observed number of operational taxonomic units (OTUs), Faith’s phylogenetic diversity and Shannon diversity. To assess beta diversity, analysis of variance (PERMANOVA) was performed using unweighted UniFrac distances. Principal-coordinate analysis (PCoA) graphs were constructed to visualize similarity between the samples. The linear discriminant analysis effect size (LEfSe) algorithm was used to identify differentially abundant taxa between the groups at genus level. For LEfSe analysis, depending on the experiments, different groups were assigned as comparison classes and were analysed by day. Briefly, in Experiment 1, RIR control and RIR AIV-infected groups were assigned as comparison classes and assessed at day 0, day 2, day 4 and day 10 post-challenge. In Experiment 2, control groups were assigned as comparison classes at days 7, 14, 21 and 28 and analysed all against all. LEfSe identified features that were statistically different between assigned groups and then compared the features using the non-parametric factorial Kruskal–Wallis sum-rank test (alpha value of 0.05) and linear discriminant analysis (LDA) cut-off value of 4.0.

## 3. Results

### 3.1. The Median Number of Operational Taxonomic Units (OTUs) Obtained from 16S rRNA Amplicon Sequencing 

All samples were normalised to 62,000 sequences per sample as they had all reached a plateau at this value, with the exception of negative control having the total number of reads generated being only 12,670 (Supplementary Figure S1). In Experiment 1, 4,235,519 total OTUs were assigned to the control group, with a median of 232,798 OTUs and 2,909,088 OTUs to the H9N2-infected group with a median of 186,513 OTUs per sample. In Experiment 2, we obtained 3,336,323 OTUs with a median of 170,859 OTUs per sample. Due to a technical issue that occurred in the sequencing stage, two samples had to be removed from the analysis (one from Experiment 1, control sample at day 0 pre-challenge, and one from Experiment 2, control at 28 days of age). Therefore, these groups had a sample size of four each in further analysis.

A total of 98% of reads obtained from the positive control (*E. coli* DH5α) were assigned to *Enterobacteriales* taxa (273,313 out of 279,747), followed by 1.7% of reads that were assigned to Bacilli. We also found *Pasteuralles*, *Betaproteobacteriales*, *Oligoflexales* and *Caulobacterales* in our positive sample. However, for each of these taxa, no more than 200 sequencing reads were assigned (which represent less than 0.07% of total reads obtained) which might be considered an Illumina error rate rather than contamination. Two taxa, Enterobacteriales and *Bacilli*, were found in the negative sample. A direct comparison between negative control and experimental samples (obtained in Experiment 1 and Experiment 2) showed that experimental samples had at least 15× to even 105× higher number of reads assigned to *Bacilli* as compared to negative control, and all experimental samples were placed above the contamination threshold (Supplementary Figure S2).

### 3.2. Alpha Diversity Indices Increased during Maturation in the Healthy URT Microbiota

**Figure 1:**
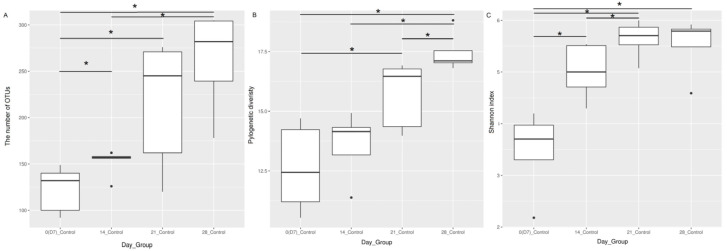
Alpha diversity indices in the chicken upper respiratory tract microbiota during maturation. The samples were collected at days (D) 7, 14, 21 and 28 of age. The alpha diversity of the upper respiratory microbiota was assessed in terms of (**A**) the number of OTUs observed, (**B**) Faith’s phylogenetic diversity and (**C**) Shannon diversity. Kruskal–Wallis pairwise testing was used to assess significant changes in the species richness of the community * *p* < 0.05.

We assessed the effect of maturation on alpha diversity in the URT microbiota at a weekly interval. The temporal changes were measured by the number of OTUs, Faith’s phylogenetic diversity and the Shannon index. Alpha diversity indices grouped by the age of the bird are shown in Figure 1. A significant increase in alpha diversity indices was seen as the chickens aged, especially between 7-day-old chicks and 3-week-old birds (Figure 1). Kruskal–Wallis statistical testing showed a statistically significant difference in the number of OTUs between samples taken at day 7 and those taken at days 14, 21 and 28 (*p* < 0.01). In addition, there was a difference in the number of OTUs observed between samples taken at day 14 and day 28 (*p* < 0.05). Changes in Faith’s phylogenetic diversity were seen between day 7 and day 21, day 14 and day 28, and day 21 and day 28 (*p* < 0.05). Shannon’s index significantly increased between day 7 and days 14, 21 and 28, suggesting that once the evenness was established at two weeks of age, it did not change significantly in the next weeks (Supplementary Table S1). 

Simple linear regression was also used to assess the association between alpha diversity indices and time (Supplementary Table S2). The number of OTUs, Faith’s phylogenetic diversity and Shannon’s diversity were all significantly correlated with time (*p* < 0.001).

### 3.3. A Significant Decrease in Alpha Diversity in Chicken URT Microbiota Was Seen at Days 2 and 10 Post H9N2 Avian Influenza Challenge

**Figure 2:**
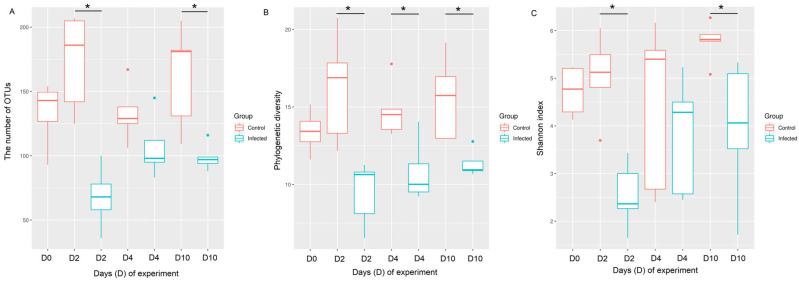
Alpha diversity indices in the chicken upper respiratory microbiota between control and H9N2-AIV-infected groups over time. Chickens were challenged with 10^5^ PFU/mL recombinant H9N2 A/chicken/Pakistan/UDL01/01. Samples were collected at day 0 pre-challenge and days 2, 4 and 10 post-challenge. (**A**) The number of OTUs observed, (**B**) Faith’s phylogenetic diversity and (**C**) Shannon diversity. Kruskal–Wallis pairwise testing was used to assess significant changes in the species richness of the community * *p* < 0.05.

The effect of H9N2 AIV infection on alpha diversity in the URT microbiota was assessed at day 2, day 4 and day 10 post-challenge (Figure 2). At days 2 and 10 post-challenge, all three alpha diversity metrics tested (the number of OTUs, Faith’s phylogenetic diversity and Shannon’s diversity) decreased in the H9N2-AIV-infected group as compared to the control group with *p* < 0.001 for all three alpha indices at day 2 post-challenge and *p* < 0.05 at day 10 post-challenge (Supplementary Table S3). Faith’s phylogenetic diversity was the only metric where the H9N2-infected group was significantly reduced compared to the control group at every time point tested (days 2, 4 and 10). The results of the Kruskal–Wallis pairwise testing are shown in Supplementary Table S3.

### 3.4. Beta Diversity Changes Are Associated with H9N2 AIV Infection

**Figure 3:**
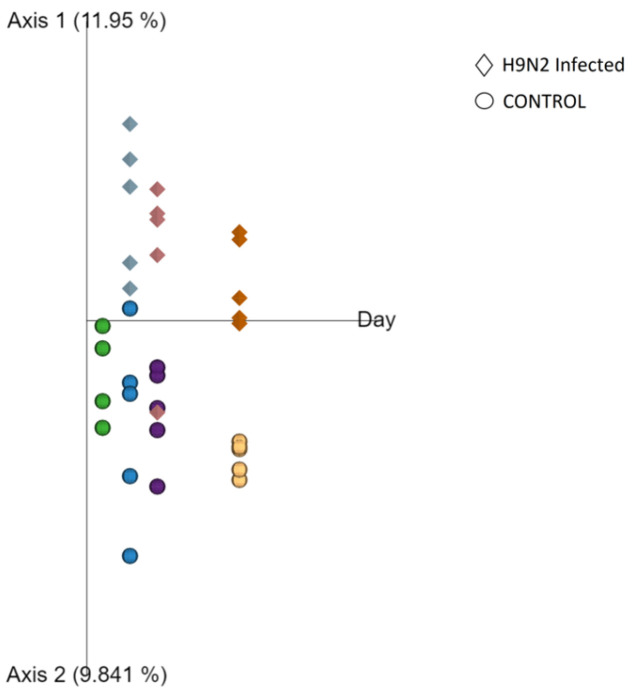
A principal coordinate analysis (PCoA) plot of the chicken upper respiratory tract microbiota following H9N2 infection using unweighted UniFrac distances. Samples collected from control group are shown as sphere objects, whereas H9N2-infected groups are shown as diamonds. The samples were collected at day 0 pre-challenge (green) and at days 2 (blue), 4 (violet) and 10 (yellow) post-challenge. The birds were challenged with 10^5^ PFU/mL recombinant H9N2 A/chicken/Pakistan/UDL01/01.

To show how beta diversity changes between groups at different time points, we performed a principal coordinate analysis (PCoA) using unweighted UniFrac distances data of taxonomic composition that include phylogenetic diversity metrics (Figure 3). PCoA plots indicate a significant separation between control and H9N2-infected chickens at all time points tested (D2, D4 and D10 post-ch). Analysis of variance (PERMANOVA) for measuring beta diversity showed that the H9N2 RIR infected group had significantly lower diversity as compared to the control group at all time points tested (*p* < 0.05 Supplementary Table S4).

PCoA was also applied to assess the changes in healthy chicken URT microbiome during maturation. A significant separation in the control groups was observed over the time of birds’ maturity (Supplementary Figure S3). Analysis of variance showed significant differences between day 7 and days 14 (*p* < 0.01), 21 (*p* < 0.05) and 32 (*p* < 0.01). In addition, significant differences were found between day 14 and day 28 (*p* < 0.01) and day 21 and day 28 (*p* < 0.05) (Supplementary Table S5). Day 14 and day 21 were the only time points that were not significantly different from one another.

### 3.5. Bacterial Taxa That Dominate the Healthy URT Microbiota during Maturation

**Figure 4:**
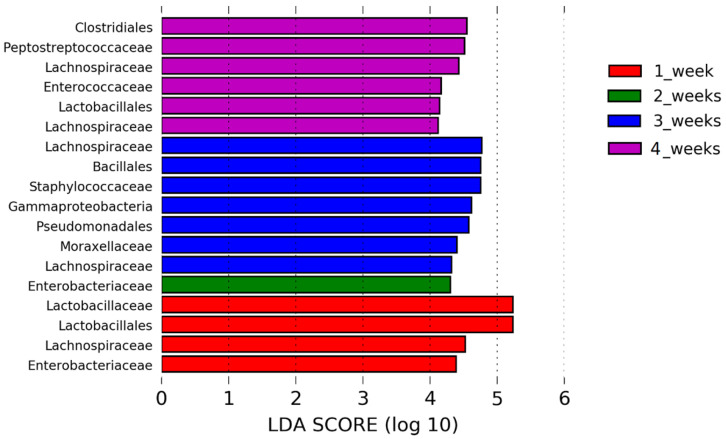
Linear discriminant analysis effect size (LEfSe) analysis identifying taxonomic differences in the upper respiratory microbiota of chickens during maturation. Histogram of LDA scores of 16S rRNA gene sequences at 1 week (red), two weeks (green), 3 weeks (blue) and 4 weeks of age (purple). Kruskal–Wallis and Wilcoxon tests were applied at alpha value 0.05. Wilcoxon test was applied among subclasses in different classes using multi-class strategy (all against all). LDA scores (log10) above 4.0 and *p* < 0.05 are shown.

The relative abundance of bacterial taxa present at the phylum, class, order and family levels was assessed to understand changes in healthy URT microbiota during maturation. The mean relative abundances of the dominant bacterial taxa that comprise the healthy respiratory microbiota from days 7 to 28 of age are shown in Supplementary Figure S4. The URT microbiota is largely characterised by *Firmicutes* and *Proteobacteria* at the phylum level and *Bacilli*, *Clostridia*, *Gammaproteobacteria* and *Actinobacteria* at the class level (Supplementary Figure S4). Interestingly, although *Bacilli* represents 30–35% of bacteria taxa in healthy trachea from day 14 onwards, the proportions of *Lactobacillacea* and *Staphylococcae* are constantly changing over time (Supplementary Figure S4). Lefse analysis indicated differences in the phylogenetic distributions of the microbiota of control chickens at the different time points at the OTU level (1 week, 2 weeks, 3 weeks and 4 weeks of age) (Figure 4). The results showed that different bacteria taxa may play a role in chicken URT maturation as we noticed that some taxa are more abundant than others depending on chicken age. The LDA scores (LDA score [log 10] > 4) indicated that the relative abundances of *Lactobacillaceae* (*Lactobacillales*) are differentially higher in young, 7-day-old chicks as compared to all other time points tested. At two weeks of age, the prevalence of *Enterobacteriaceae* was enriched, whereas at three weeks of age, the most differentially abundant bacterial taxon was characterised by a preponderance of *Bacillales* (*Staphylococcae*), *Lachnospiraceae*, *Gammaproteobacteria* (*Pseudomonales*) and *Actinobacteria*. *Clostridiales* (*Peptostreptococceae*, *Lechnospiraceae*) and *Lactobacillales* were much more enriched at four weeks of age as compared to any other time tested. In addition, we performed a two-tailed *t*-test analysis of changes at the phylum, class, order and family levels between each of the time points tested (Supplementary Table S6). At the order level, there was a significantly higher relative abundance of *Lactobacillales* when comparing samples from day 7 to day 14, day 14 to day 21, and day 21 to day 28 (*p* < 0.01). We also noticed a significant increase in the *Clostridiales* family when comparing samples from day 7 to day 28 (*p* < 0.05) and day 14 to day 28 (*p* < 0.01).

### 3.6. Lactobacillaceae (Lactobacillus) Was the Most Differentially Abundant Taxa in URT Microbiota following H9N2 AIV Infection

**Figure 5:**
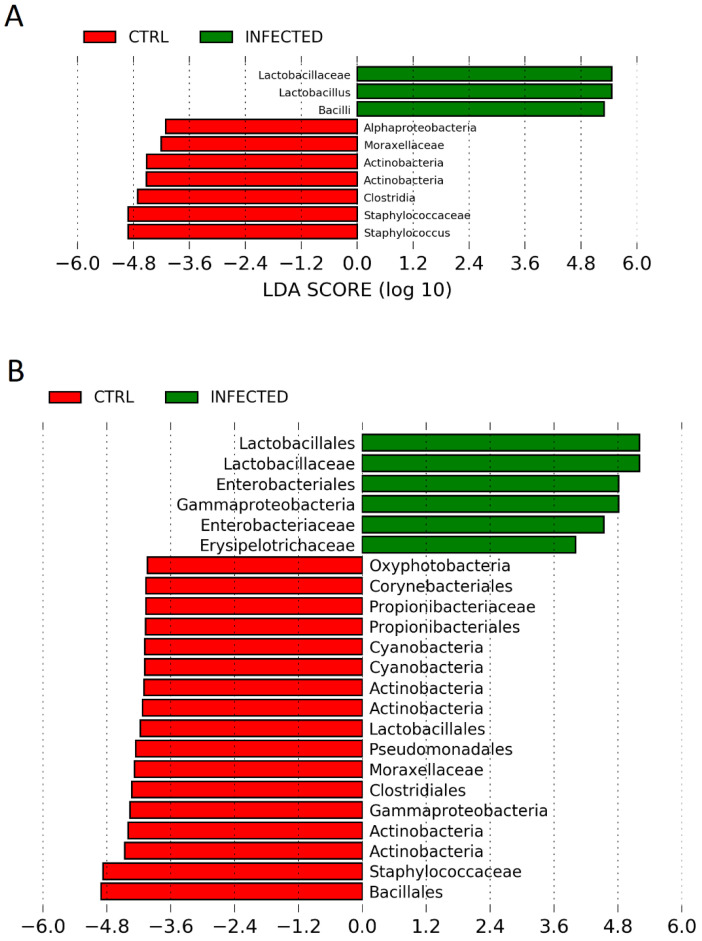
Linear discriminant analysis effect size (LEfSe) analysis identifying taxonomic differences in the upper respiratory microbiota of chickens infected with H9N2 avian influenza virus. Histogram of LDA scores of 16S rRNA gene sequences at day 2 post-challenge (**A**) and day 10 post-challenge (**B**). Control birds are represented by red, whereas infected birds are represented by green colour. The birds were challenged with 10^5^ PFU/mL recombinant H9N2 A/chicken/Pakistan/UDL01/01. Kruskal–Wallis and Wilcoxon tests were applied at alpha value 0.05. LDA scores (log10) above 4.0 and *p* < 0.05 are shown.

We aimed to assess whether infection with H9N2 AIV causes the composition of URT microbiota to be dysregulated and whether certain bacterial taxa are associated with the acute or recovery phase of infection. The relative abundance of bacterial taxa present at the phylum, class, order and family levels in control and infected chickens is shown in Supplementary Figure S5. LEfse analysis indicated differences in the bacterial groups identified in the microbiota of H9N2-infected and control chickens at different time points tested (Figure 5). The LDA scores indicated that the relative abundances of Bacilli (*Lactobacillus*) were much more enriched in H9N2-infected birds versus control at day 2 post-challenge (Figure 5A) (LDA score [log 10] > 4). Additionally, two-tailed *t*-test analysis of changes at the phylum level showed significant growth in the relative proportion of *Firmicutes* at day 2 post-ch in the infected group compared to the control group due to the significant increase in the relative abundance of the family *Lactobacillaceae* (as well as its class *Bacilli* and order *Lactobacillales*), as this family highly dominates the respiratory microbiota at this time point (Supplementary Table S7). The most differentially abundant bacterial taxon in control birds was characterised by a preponderance of *Staphylococcaceae* (*Staphylococcus*) and *Actinobacteria* (LDA score [log10] > 4) at day 2 post-challenge. 

Furthermore, we observed a differential abundance of bacterial taxa between H9N2-infected and control birds at day 10 post-ch. The LDA scores indicated that the relative abundances of *Lactobacillaceae* (*Lactobacillales*) and *Enterobacteriaceae* (*Enterobacteriales*) were much more enriched in H9N2-infected birds versus control at the recovery phase of infection (Figure 5B) and the most differentially abundant bacteria taxa (LDA score [log 10] > 4.0). A significant increase in the relative abundance of the family *Lactobacillaceae* at day 10 post-ch was also seen by two-tailed *t*-test analysis (Supplementary Table S7). The control birds were characterised mainly by a preponderance of *Bacillales* (*Staphylococcaceae*), *Actinobacteria*, *Gammaproteobacteria*, *Propionibacteriaceae* and *Corynebacteriales*. No differentially abundant taxa were seen above LDA score [log10] > 4 at day 4 post-challenge besides *Enterobacteriales* enriched in H9N2-infected birds (Supplementary Figure S6). 

### 3.7. The Influenza A Virus Matrix Gene Is Detected at Days 2 and 4 Post-Challenge in the Tracheas of the H9N2 AIV Chickens

We performed qRT-PCR on RNA extracted from H9N2-AIV-infected trachea samples using primers specific for the M-gene to verify that the virus is replicating in the respiratory tract. Copies of the M-gene were detected in all samples tested at days 2 and 4 post-challenge (Supplementary Figure S7).

### 3.8. H9N2 AIV Infection Is Associated with Increased Damage to the Inner Epithelial Cell Layer in Tracheas at Day 4 but Not at Day 2 and Day 10 Post-Challenge 

**Figure 6:**
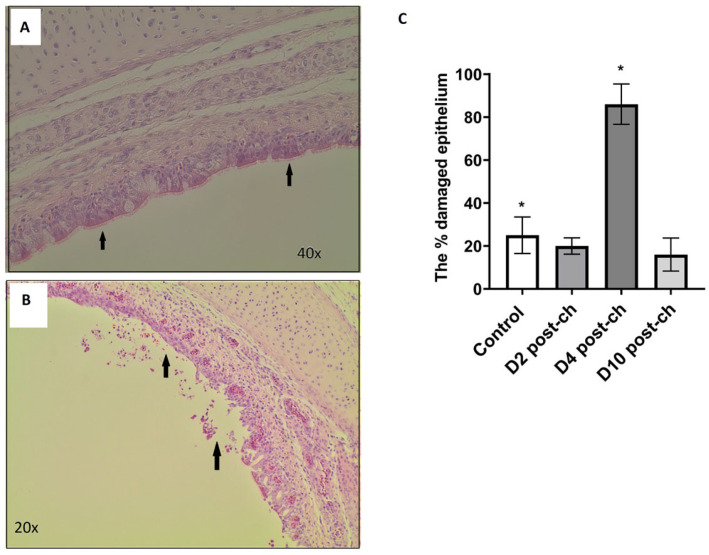
Histological examination of the control and infected tracheas. Cross sections of trachea samples from control chickens and chickens infected with H9N2 AIV A/chicken/Pakistan/UDL01/08 stained with haematoxylin and eosin (**A**,**B**). (**A**) Healthy section of the inner epithelial layer (arrows); (**B**) section of the inner epithelial layer that has degraded (arrows). Samples collected at day 4 post-challenge. Images were captained with an imaging microscope (EVOS XL Core Imaging System, Thermo Fisher). (**C**) Estimated percentage damage to the inner epithelial layer in tracheas of control and H9N2-AIV-infected chickens. Control group is represented by six samples that were taken from birds at 2–10 days of experiment, whereas samples from infected birds (N = 5 each) were collected at days 2, 4 and 10 post-challenge (post-ch). The percentage of the inner epithelial layer that had either been degraded or had the cilia on its surface stripped away was estimated independently by two people, and the mean percentage damage of the two estimates was used in the statistical analysis. Two-tailed *t*-tests were used to assess significant differences between the control and infected groups * *p* < 0.05.

To assess the damage to the inner epithelial layer of the respiratory tract, samples of the tracheas were taken at days 2, 4 and 10 post-ch with H9N2 AIV from infected birds and were examined for inner epithelial cell layer damage. Control samples were collected from a range of birds on days 2–10. As compared to the healthy inner epithelial layer of the trachea section (6A), the damage appeared to occur in two ways: the degeneration of the epithelial layer (Figure 6B) or where the cilia on the cell surface were damaged, but the epithelial layer was left intact. Independent estimates were made for the percentage of the inner epithelial layer that was damaged (Figure 6C). Two-tailed *t*-tests were used to assess where there were significant differences between the control and infected groups. At days 2 and 10 post-challenge, no differences were shown between the control and infected groups (*p* = 0.6288 and *p* = 0.4605, respectively). The H9N2-infected group at day 4 post-challenge showed significantly more damage to the inner epithelial layer (*p* = 0.0009) as compared to the control group. It is, however, worth noticing that we also observed substantial damage to the tissues in the control samples collected at day 4 post-ch (N = 2) which was not seen at day 2 and day 10 post-ch at the control groups, which contributed to the average epithelium damage seen in the control group (Figure 6C).

## 4. Discussion

We demonstrated that the chicken upper respiratory (URT) microbiota is altered at the acute and recovery phases of AIV H9N2 infection. Both, alpha and beta diversity indices were continuously compromised following AIV infection. Furthermore, the composition of bacteria taxa also differs, suggesting that different bacteria taxa might play a role in the acute and recovery phases from AIV infection. The presence of the viral M-gene in the tracheas of chickens infected with H9N2 AIV was seen during the acute, infectious phase (days 2 and 4 post-challenge), verifying the replication of influenza A virus in tracheas at the time points tested. In addition, the highest levels of oral viral shedding were also detected at day 2 post-challenge which ceased by day 6 [19], providing further evidence of active infection. During the acute phase of infection, the most significant losses in species richness, evenness and phylogenetic diversity were seen, which continued to be compromised also at the recovery phase of infection. In our previous study [19], we saw a similar trend where all colon microbiota of H9N2-AIV-infected birds lost their overall richness at the acute phase of infection; however, species richness was restored in the recovery phase through the predominant bacteria becoming more dominant within the microbiota which we did not see in URT microbiota. Therefore, the typical levels of diversity in the upper respiratory microbiome are not quickly restored after the infectious phase. 

The major bacterial families that characterise the healthy upper respiratory microbiota during maturation are *Lactobacillaceae*, *Staphylococcaceae*, *Lachnospiraceae*, *Enterobacteriaceae* and *Ruminococcaceae*. This is in agreement with previous studies, which have shown that members of *Lactobacillales* are the most abundant in the respiratory microbiota, along with others like *Enterobacteriales* [20,26,27]. It has also been shown previously that *Lactobacillaceae* members are early colonisers of the chicken respiratory tract but become less abundant as the microbiota becomes more diverse [20]. This is supported in our findings, as *Lactobacillaceae* is the most abundant at 7 days of age, but it becomes less prominent over time as species evenness advances over time. However, the composition of bacteria in the URT can change during the H9N2 AIV infection as we showed in this study. In the acute phase of H9N2 AIV infection, *Lactobacillaceae* highly dominates the upper respiratory microbiota. Furthermore, *Lactobacillaceae* were still predominant bacteria at the recovery phase of infection suggesting an important role of this family in surviving influenza infection. *Lactobacillaceae* is a family of Gram-positive lactic acid bacteria that has been shown to act as a probiotic [28]. Members of the *Lactobacillaceae* family, particularly the genus *Lactobacillus*, have been shown to regulate the immune system and can activate innate and adaptive immune responses [28,29]. Interestingly, in our previous study on the colon microbiota, we noticed that although the *Firmicutes* phylum was the most differentially abundant between infected and non-infected individuals, the *Lactobacillales* was missing in the colon at the recovery phase of infection, and this trend was seen in two genetically distinct chicken breeds infected with H9N2 AIV [19]. The exact reason why *Lactobacillaceae* is so enriched in the acute and recovery phases of H9N2 AIV infection in the chicken upper respiratory tract and missing at the recovery phase in colon microbiota needs to be further investigated. However, this is the first report showing that there might be some potential interactions and cross-talk between gut-respiratory microbiota during active viral infection in chickens. 

The next question we asked in this study was whether the reason for the dysbiosis of the respiratory microbiota observed during the H9N2 AIV infection relates to the physical disruption of the epithelial cell lining of the respiratory tract, which in turn could potentially cause the dysbiosis of the microbial communities that reside on it. No difference in epithelial cell lining was observed between the control and infected groups at days 2 and 10 post-challenge, suggesting that there must be another factor/s that corresponded to microbiota dysbiosis at this time point tested. We hypothesised that the reason for dysbiosis particularly at the acute phase of infection might be connected to an innate immune response elicited towards the replicating influenza A virus which could potentially directly impair the presence of some commensal bacteria, or it might have created an inflammatory environment where only some types of bacteria can survive. This suggests that bacteria might be either killed directly by immune responses or bacteria that are better adapted to persist in harsher conditions are able to thrive, while other species die out. The association between damage to the inner epithelial layer and H9N2 AIV infection was seen at day 4 post-challenge; however, this is not substantially associated with differences in microbial composition between the control and infected chickens observed in this study. The damage to the tissues in infected samples could be connected to the antiviral mechanism regulated by an interplay between different cytokine and interferon types. Morris et al. (2023) showed the upregulation of genes responsible for defence and inflammatory responses at day 5 in lungs following the H9N2 challenge in chickens [30]. 

In addition to assessing changes in the URT microbiome, during the AIV H9N2 infection, we also showed that one of the factors that determines the composition of the healthy respiratory microbiota in the URT is age, as we observed alpha diversity indices increasing over time and differential enrichment of bacterial taxa as chickens matured up to 28 days of age. A significant positive correlation between alpha diversity indices and increasing age was observed, with the most significant period being between day 7 and days 14, 21 and 28. Beta diversity analysis supported our finding that microbial diversity is shaped by ageing. Interestingly, no significant changes in beta diversity were found between days 14 and 21 of age or in alpha diversity indices (OTUs and phylogenetic diversity) at this time point tested, indicating that the least substantial changes occur between weeks two and three of age. Although our control Experiment 2 finished at the bird age of 28 days, we observed significant separation between day 0 and 10 control groups in our challenge experiment (Experiment 1). This provides further evidence for age being a factor that influences the respiratory microbiota, as it suggests that the microbial composition in the respiratory tract significantly evolved during maturation from days 21 to 31 of age, which goes further beyond the timeframe of age we studied in chickens for Experiment 2. It was previously shown that the respiratory tract of chickens is populated by a diverse community of commensal bacteria, whose composition can be altered by a number of host- and environment-related factors [20,22,26]. 

In conclusion, this is the first study showing alteration of upper respiratory tract microbiota flowing the H9N2 AIV infection in chickens. We observed that alpha and beta diversity in the respiratory microbiota changes in both the acute and recovery phases of influenza A virus infection and that there is dysbiosis in the abundance of different bacterial taxa at different time points. A lack of homeostasis in the upper respiratory tract seems to be connected to different factors and might change over the time of infection. Dysbiosis might be more associated with anti-viral response, which could then directly or indirectly (by changing the environment) affect microbiota on mucosal surfaces, and less with physical damage caused to the inner epithelial layer cells by the replicating virus. The family *Lactobacillaceae* is most strongly associated with infected chickens compared to the controls during the acute phase and continues to be a major player in the respiratory microbiota into the recovery phase, while the abundance of less dominant families is reduced to levels lower than in control chickens. Although the reason why the *Lactobacillaceae* family was the main player during the AIV infection in the upper respiratory tract of chickens is unknown and needs to be investigated further, this study might suggest that supplementation with *Lactobacillaceae* could potentially aid recovery from H9N2 influenza infection. 

## Data Availability

Raw Illumina sequencing reads are available upon request.

## Supplementary Materials

The following supporting information can be downloaded at https://www.mdpi.com/article/10.3390/pathogens12091168/s1, Supplementary Table S1: Kruskal–Wallis pairwise testing of the association between alpha diversity and age in the healthy respiratory microbiota in chickens, Supplementary Table S2: Simple linear regression of the association between alpha diversity in the respiratory microbiota and the age of chickens, Supplementary Table S3: Kruskal–Wallis pairwise testing of the differences in alpha diversity between the control and H9N2-AIV-infected respiratory microbiota in chickens, Supplementary Table S4: Analysis of variance (PERMANOVA) of beta diversity between the healthy respiratory microbiota at different ages, Supplementary Table S5: Analysis of variance (PERMANOVA) of beta diversity between control and H9N2-AIV-infected respiratory microbiota, Supplementary Table S6: Two-tailed *t*-test analysis of changes in relative abundances of the dominant bacterial taxa in the healthy respiratory microbiota during maturation, Supplementary Table S7: Two-tailed *t*-test analysis of changes in relative abundances of the dominant bacterial taxa in the respiratory microbiota between control and H9N2-AIV-infected groups at different time points, Supplementary Figure S1: Rarefaction curve for the observed number of OTUs, Supplementary Figure S2: The number of reads assigned to Bacilli per sample, Supplementary Figure S3: A principal coordinate analysis (PCoA) plot of the healthy chicken upper respiratory tract microbiota categorised by day using unweighted UniFrac distances, Supplementary Figure S4: Bacterial taxa that dominate the healthy URT microbiota during maturation, Supplementary Figure S5: Bacterial taxa associated with H9N2 infection in the respiratory microbiota, Supplementary Figure S6: Linear discriminant analysis effect size (LEfSe) identifying differences between H9N2-AIV-infected chickens and controls at day 4 post-challenge, Supplementary Figure S7: The number of copies per millilitre of the influenza A virus matrix gene (M-gene) detected in trachea samples taken from chickens at days 2 and 4 post-challenge with H9N2 AIV A/chicken/Pakistan/UDL01/08 by qRT-PCR.
