# Supplementary material for: Alteration of the Chicken Upper Respiratory Microbiota, Following H9N2 Avian Influenza Virus Infection
Source: Pathogens. 2023 Sep 16;12(9):1168. doi: 10.3390/pathogens12091168 (PMC10534358; doi:10.3390/pathogens12091168)
Supplement: Supplementary file 1 [file pathogens-12-01168-s001.zip › Suppl. Tables_Microbiota_ChrzastekK.pdf]

Supplementary (S) Tables

S1. Kruskal Wallis pairwise testing of the association between alpha diversity and age in the healthy respiratory microbiota in chickens. Kruskal Wallis pairwise testing was used to assess the changes in alpha diversity indices between time points in Experiment 2. Samples in Experiment 2 were taken at 7, 14, 21 and 28 days of age from healthy RIR chickens. The alpha diversity in the respiratory microbiota was tested using the number of OTUs detected, Faith's phylogenetic diversity and Shannon diversity.

Alpha Diversity	Group 1	Group 2	H	p-value	q-value
The number of OTUs	Day 7	Day 14	3.93818182	0.04720177	0.11981987
	Day 7	Day 21	3.93818182	0.04720177	0.11981987
	Day 7	Day 28	6	0.01430588	0.05974321
	Day 14	Day 21	2.15121951	0.1424567	0.21984893
	Day 14	Day 28	6	0.01430588	0.05974321
	Day 21	Day 28	1.5	0.22067136	0.29723081
Faith's Phylogenetic Diversity	Day 7	Day 14	0.88363636	0.34720764	0.44932753
	Day 7	Day 21	3.93818182	0.04720177	0.12461267
	Day 7	Day 28	6	0.01430588	0.06721111
	Day 14	Day 21	3.15272727	0.07580017	0.16676038
	Day 14	Day 28	6	0.01430588	0.06721111
	Day 21	Day 28	4.86	0.02748634	0.07777034
Shannon Diversity	Day 7	Day 14	6.81818182	0.00902344	0.08507814
	Day 7	Day 21	6.81818182	0.00902344	0.08507814
	Day 7	Day 28	6	0.01430588	0.08961482
	Day 14	Day 21	3.93818182	0.04720177	0.15013056
	Day 14	Day 28	2.16	0.14164469	0.26259733
	Day 21	Day 28	0	1	1

S2. Simple linear regression of the association between alpha diversity in the respiratory microbiota and the age of chickens. Alpha diversity metrices (the number of OTUs, Faith's phylogenetic diversity and Shannon Diversity) were used as the dependent variable in simple linear regression to determine whether there is correlation between the species richness in the respiratory microbiota and chicken age. Samples tested were taken at 7, 14, 21 and 28 days of age.

**Alpha Diversity**	**R2**	**Slope (Y value)**	**Equation**	**P Value**
Observed Number of OTUs	0.5998	1.356	Y = 6.844*X + 67.85	<0.0001
Faith's Phylogenetic Diversity	0.6538	0.04167	Y = 0.2361*X + 10.70	<0.0001
Shannon Diversity	0.5684	0.02132	Y = 0.1009*X + 3.167	0.0002

S3. Kruskal Wallis pairwise testing of the differences in alpha diversity between the control and H9N2 AIV infected respiratory microbiota in chickens. Kruskal Wallis pairwise testing was used to assess how alpha diversity indices change between control groups and groups challenged with H9N2 AIV A/chicken/Pakistan/UDL01/08 at days 2 (D2), 4 (D4) and 10 (D10) post-challenge in RIR chickens (Experiment 1). The alpha diversity in the respiratory microbiota was tested using the number of OTUs detected, Faith's phylogenetic diversity and Shannon diversity.

Alpha Diversity	Days Post-Challenge	H	p-value	q-value
The number of OTUs	D2	6.81818182	0.00902344	0.05974321
	D4	2.45454546	0.11718509	0.21483933
	D10	5.77090909	0.0162936	0.05974321
Faith's Phylogenetic Diversity	D2	6.81818182	0.00902344	0.06721111
	D4	4.81090909	0.02828012	0.07777034
	D10	6.81818182	0.00902344	0.06721111
Shannon Diversity	D2	6.81818182	0.00902344	0.08507814
	D4	0.88363636	0.34720764	0.44932753
	D10	4.81090909	0.02828012	0.10979342

S4. Analysis of variance (PERMANOVA) of beta diversity between the healthy respiratory microbiota at different ages. To assess beta diversity between different time points in Experiment 2, unweighted UniFrac distances were used in PERMANOVA. Samples in Experiment 2 was taken at 7, 14, 21 and 28 days of age.

Group 1	Group 2	pseudo-F	p-value	q-value
Day 7	Day 14	1.843418132	0.008	0.022578947
Day 7	Day 21	2.455765667	0.012	0.022578947
Day 7	Day 28	3.697054644	0.005	0.022578947
Day 14	Day 21	1.28880597	0.052	0.073021277
Day 14	Day 28	2.580119787	0.006	0.022578947
Day 21	Day 28	1.821964447	0.026	0.039906977

S5. Analysis of variance (PERMANOVA) of beta diversity between control and H9N2 AIV infected respiratory microbiota. To assess beta diversity between different time points in Experiment 1, unweighted UniFrac distances were used in PERMANOVA. Samples were taken at day 0 (D0) pre-challenge and at days 2 (D2), 4 (D4) and 10 (D10) post-challenge in control chickens and chickens challenged with H9N2 AIV A/chicken/Pakistan/UDL01/08.

Group 1	Group 2	pseudo-F	p-value	q-value
CTRL D0	CTRL D2	1.12756	0.208	0.2288
CTRL D0	Infected D2	1.955375	0.009	0.022578947
CTRL D0	Infected D4	2.002263	0.028	0.042
CTRL D0	Infected D10	1.750084	0.006	0.022578947
CTRL D2	Infected D2	2.075569	0.005	0.022578947
CTRL D2	Infected D4	1.899262	0.03	0.043043478
CTRL D2	Infected D10	1.712934	0.015	0.025384615
CTRL D4	Infected D4	1.884073	0.026	0.039906977
CTRL D4	Infected D10	1.580944	0.009	0.022578947
CTRL D10	Infected D10	2.146785	0.012	0.022578947

S6. Two-tailed t-test analysis of changes in relative abundances of the dominant bacterial taxa in the healthy respiratory microbiota during maturation. Two-tailed t-tests were used to assess how the relative abundances of bacterial taxa at phylum, class, order and family level change between different ages during maturation. Samples of the respiratory microbiota were taken at day 7 (D7), 14 (D14), 21 (D21) and 28 (D28). Only bacterial taxa that accounted for more than 1% of total sequences were included in the graphs. All other bacterial taxa were categorised as 'Other'.

**Bacterial Taxa**	**Group Pairings**					
	**D7 D14**	**D7 D21**	**D7 D28**	**D14 D21**	**D14 D28**	**D21 D28**
**Phylum Level**						
Actinobacteria	0.070479	0.050977	0.23694	0.710197	0.462505	0.315832
Firmicutes	0.067229	0.162042	0.878503	0.582231	0.028109	0.081141
Proteobacteria	0.094927	0.195898	0.810353	0.563305	0.038984	0.075587
Other	0.257428	0.963172	0.936832	0.203231	0.229532	0.880739
**Class Level**						
Actinobacteria	0.063836	0.049891	0.241134	0.744997	0.414006	0.298763
Bacilli	0.253242	0.052482	0.157876	0.180543	0.546244	0.427871
Clostridia	0.341719	0.557329	0.042995	0.195407	0.00154	0.318713
Alphaproteobacteria	0.006441	0.03074	0.166074	0.673572	0.433652	0.358379
Gammaproteobacteria	0.112765	0.241603	0.740325	0.505886	0.039521	0.072502
Other	0.857599	0.817597	0.432822	0.995679	0.557588	0.454002
**Order Level**						
Corynebacteriales	0.189263	0.153678	0.373971	0.728018	0.321629	0.315377
Propionibacteriales	0.10657	0.046058	0.369735	0.73526	0.508449	0.325371
Bacillales	0.001022	0.009621	0.010299	0.126793	0.141482	0.075967
Lactobacillales	0.104493	0.006265	0.098038	0.019124	0.747027	0.026919
Clostridiales	0.341719	0.557329	0.042995	0.195407	0.00154	0.318713
Betaproteobacteriales	0.314155	0.221674	0.969566	0.395285	0.371286	0.283636
Enterobacteriales	0.221997	0.857193	0.761448	0.174287	0.10803	0.408671
Pseudomonadales	0.137666	0.06184	0.73149	0.202939	0.100799	0.080402
Other	0.002262	0.039035	0.238429	0.629033	0.630406	0.49778
**Family Level**						
Corynebacteriaceae	0.189263	0.158282	0.373971	0.722767	0.321629	0.322238
Propionibacteriaceae	0.12948	0.03166	0.337235	0.869919	0.575345	0.40401
Staphylococcaceae	0.0002	0.008229	0.023666	0.09715	0.080057	0.059108
Lactobacillaceae	0.082136	0.005879	0.064225	0.017344	0.534994	0.063102
Family XI	0.356339	0.983505	0.456621	0.357042	0.376897	0.375385
Lachnospiraceae	0.079583	0.37311	0.056789	0.081177	0.001582	0.704507
Ruminococcaceae	0.196306	0.467582	0.759995	0.108931	0.005192	0.354574
Burkholderiaceae	0.311983	0.223401	0.86452	0.399149	0.20495	0.240814
Enterobacteriaceae	0.221997	0.857193	0.761448	0.174287	0.10803	0.408671
Moraxellaceae	0.270612	0.066002	0.654778	0.194091	0.193102	0.078969
Pseudomonadaceae	0.052892	0.197058	0.276808	0.722791	0.153514	0.316791
Other	0.006566	0.092666	0.000227	0.74573	0.004502	0.010244

S7. Two-tailed t-test analysis of changes in relative abundances of the dominant bacterial taxa in the respiratory microbiota between control and H9N2 AIV infected groups at different time points. Two-tailed t-tests were used to assess how the relative abundances of bacterial taxa at phylum, class, order and family level change between control chickens and chickens challenged with H9N2 AIV, compared at the same time point tested. Samples of the respiratory microbiota were taken at days 2 (D2), 4 (D4) and 10 (D10) post-challenge in both groups. Only bacterial taxa that accounted for more than 1% of total sequences were included in the graphs. All other bacterial taxa were categorised as 'Other'.

**Bacterial Taxa**	**Days Post-Challenge**		
	**D2**	**D4**	**D10**
**Phylum Level**			
Actinobacteria	0.101986	0.363521	0.02832
Firmicutes	0.031744	0.957259	0.461521
Proteobacteria	0.099331	0.584721	0.723955
Other	0.713574	0.121416	0.008364
**Class Level**			
Actinobacteria	0.102668	0.368062	0.023773
Bacilli	0.013956	0.96375	0.314503
Clostridia	0.1374	0.984636	0.459807
Alphaproteobacteria	0.085207	0.709262	0.255356
Gammaproteobacteria	0.147105	0.54864	0.505935
Other	0.980731	0.132289	0.079513
**Order Level**			
Corynebacteriales	0.016625	0.684078	0.004009
Propionibacteriales	0.036689	0.229746	0.01436
Bacillales	0.070597	0.449441	0.012673
Lactobacillales	0.00436	0.86831	0.059004
Clostridiales	0.1374	0.984636	0.459807
Betaproteobacteriales	0.082501	0.576468	0.353347
Enterobacteriales	0.233271	0.223211	0.050248
Pseudomonadales	0.951982	0.642295	0.045553
Other	0.195132	0.158532	0.083361
**Family Level**			
Corynebacteriaceae	0.020438	0.679493	0.004705
Propionibacteriaceae	0.046992	0.237754	0.016283
Staphylococcaceae	0.067708	0.43341	0.01497
Lactobacillaceae	0.004232	0.827726	0.042089
Family XI	0.336359	0.641122	0.103631
Lachnospiraceae	0.23259	0.358378	0.885705
Ruminococcaceae	0.168313	0.320158	0.841101
Burkholderiaceae	0.116916	0.56359	0.496488
Enterobacteriaceae	0.233271	0.223211	0.050248
Moraxellaceae	0.03933	0.92905	0.130859

Pseudomonadaceae	0.485736	0.097844	0.384672
Other	0.082999	0.169418	0.029697
